# Hospital and Institutional News

**Published:** 1912-05-04

**Authors:** 


### HOSPITAL AND INSTITUTIONAL NEWS.

### THE KING AND THE LEAGUE OF MERCY.

THEIR Majesties' attendance at the London Opera House last Monday is only the latest example of their keen interest in that side of hospital work for which the League of Mercy is responsible. The matinée, we may add, was a double success. Not merely was the sum of rather more than £1,000 realised for the funds of the League, but its claim upon fashionable society was once more asserted and enforced. Such festival occasions as this matinée, for which the generous support of the world of the theatre cannot be thanked too sincerely, have their value in reminding the public that the League of Mercy has succeeded in dovetailing the efforts of the rich and the poor, and of demanding from the former an amount of personal service which is invaluable to the voluntary hospitals. We are glad to note that the League of Mercy was introduced into the programme, and Mr. G. R. Sims' Ode as recited by Sir George Alexander was a valuable reminder of the purpose of the matinée. The King and Queen were received by Prince and Princess Alexander of Teck, the Grand President and Lady Grand President of the League, Princess Victoria of Schleswig-Holstein, Dora Countess of Chesterfield, the Earl of Chesterfield, Lord and Lady Farquhar, and Mrs. Ronalds. The Queen accepted a bouquet from Miss Doria Stanhope, and Princess Alexander of Teck was presented with one by Miss Ines Clark.

### THE SHEFFIELD ROYAL HOSPITAL.

DURING Sir Henry Burdett's absence abroad the Report of a Special Committee of Investigation relating to his Report on the Sheffield Royal Hospital (see THE HOSPITAL, February 10, 1912, page 487) was presented to the quarterly meeting. No copy of this Report was sent to Sir Henry Burdett, and on his writing for a copy the Secretary replied that the request would be laid before the board of management. No copy has so far reached him in reply to his request. Though the Report appears to have been sent generally to the Press, several newspapers having published extracts from it, we have not seen it, and therefore have not dealt with it because we do not know its contents.

### "HOSPITAL CRITICISM."

UNDER this heading our contemporary *The Builder* has an article in its issue of 26th ult. relating to the criticisms of the Sheffield Royal Hospital. *The Builder* accepts the Report of the Investigation Committee without question, and bases upon it the conclusion that "a layman's opinion can hardly be accepted as the last word on hospital construction as distinct from its administration, nor as to the best way to lay out a hospital on a given site to avoid waste of space and subsequent cost of upkeep." We would venture to suggest that *The Builder* should obtain complete plans of the whole of the buildings of the Royal Hospital, Sheffield, and publish them. Then we have little doubt that the views of our contemporary as expressed in its issue of the 26th ult. will necessarily be materially modified. We may remind *The Builder* and our contemporaries of the lay Press, that Sir Henry Burdett's inspections of hospitals throughout the country have been undertaken with a view to bring out clearly how far each institution reaches the standard of requirements of an up-to-date modern hospital. It is this fact which constitutes much of their value, and its recognition has caused hospital authorities generally to realise that the writer's object from first to last has been to help to raise the standard of efficiency of our voluntary hospitals everywhere.

### THE SELECT COMMITTEE ON SECRET REMEDIES.

THE Select Committee on Secret Remedies has now been appointed, and includes among its fifteen members those whose names were mentioned in last week's HOSPITAL as having been approached by the Home Office with reference to the matter. The full Committee is composed as follows: Mr. Charles Bathurst, Mr. Cawley, Dr. Chapple, Sir Henry Dalziel, Mr. Marshall Hall, Mr. Hayden, Mr. Hodge, Mr. Ingleby, Mr. Glyn-Jones, Mr. Haydn Jones, Mr. Lawson, Mr. Lynch, Sir Philip Magnus, Mr. Newton, and Sir Henry Norman. The last named will probably be appointed chairman, and five will form a quorum. Two of the members, Dr. Chapple and Mr. Lynch, are registered medical men; two others, Mr. Dalziel and Mr. Harry Lawson, are newspaper proprietors; and one, Mr. Glyn-Jones, is a registered pharmacist and Parliamentary Secretary of the Pharmaceutical Society. Classified politically, six of the members are Liberals, six Conservatives, two Nationalists, and one is a Labour member.

### THE DEFENCE OF PATENT MEDICINES.

As was mentioned in a recent article in THE HOSPITAL, the proprietors of secret remedies, who are well organised, have taken great pains to prepare their defence. One of their organisations, the "Owners of Proprietary Articles" sub-section of the Chemical section of the London Chamber of Commerce, has caused to be issued a pamphlet which is apparently designed to prejudice the inquiry. The pamphlet, in which it is declared that the agitation has been mainly instigated by medical men who desire to extend their monopoly, claims that there is no evidence of special injury to the public from the sale of proprietary medicines and goods. This pamphlet is not likely to improve the case for the manufacturers in view of the circumstances under which it is issued. It will be for the Committee to decide whether or not there is evidence that injury is caused by the use of nostrums.

### SAVAGE ASSAULT ON A DOCTOR.

MR. MARSHALL JOHN CAMPBELL, a Liverpool practitioner, has been the victim of a particularly ferocious assault. Last Saturday night he received an urgent call to attend a woman patient, whom the messenger, a boy, declared to be seriously ill. On arriving at the house Mr. Campbell was received by a seeming workman. The house was in complete darkness and appeared to be uninhabited. The man asked the doctor to wait while he fetched a light, and in his absence, without any warning, blows were suddenly rained upon the doctor's head from behind by a couple of men. Falling to the ground, but not losing consciousness, he had time to see one of his assailants rush out of the front door. A crowd quickly gathered and Mr. Campbell was assisted home. He is unable to suggest any other motive for this assault except robbery. He was wearing a diamond pin, two valuable rings, and a gold watch and chain at the time. Two arrests have been made by the police. The most dastardly feature in the affair is the use which was made of the humanity of the profession, and the ruse employed to draw the victim into a deserted house. The claim of urgency has often been abused before now by ignorance or selfishness, but it has been left to a Liverpool desperado to make it an excuse for a brutal assault on a practitioner whose years alone should command respect and consideration. Fortunately Mr. Campbell is understood to be progressing favourably.

### WIMBLEDON'S NEW HOSPITAL OPENS.

This week the new hospital at Copse Hill, Wimbledon—which is not to be confounded with the Nelson Hospital at Merton, South Wimbledon—has started work. The architect is Mr. T. G. Jackson, and, it should be recalled, the cost has received a contribution of £1,030 from King Edward's Hospital Fund. The theatre has been largely paid for by the Collyns Memorial Fund, which has been raised in memory of the late Dr. Collyns, a medical practitioner of the district. The hospital has now thirty-two beds, including a children's ward with four beds. As regards the wards, the men's and the women's each accommodate twelve patients, beside which there are two single-bedded wards for serious cases, and one for private patients which will be available at a fee of 25s. per week. Little remains but the slope of the site to recall the institution as it was prior to the present rebuilding. How extensive that enlargement has been may be judged from the fact that the accommodation was originally for sixteen only.

### THE SCHIFF HOME OF RECOVERY.

The administration of the Home at Knowle Hill Park, Cobham, which is known by the name of its munificent founder, Sir Ernest Schiff, has now been systematised by a scheme prepared by the Charity Commissioners. The object in view when the charity was instituted was the provision of a healthy free home for poor persons of either sex recovering from the effects of accident or operations and still in need of surgical supervision and nursing. It was also stipulated that the benefits of the home should be confined to those who have been in-patients at one or other of seven general hospitals: Charing Cross, Guy's, King's College, London, Middlesex, St. Thomas's, and University College. No person is to be received who is suffering from inoperable malignant disease, epilepsy, phthisis, or any other disease that the medical committee shall from time to time determine to exclude. With regard to this provision, with the object of which no reasonable person can quarrel, the exact meaning of the expression "suffering from" is rather uncertain. In practice it will probably only be utilised to exclude those definitely likely to exhibit epileptic stigmata or to be a source of tuberculous infection to others.

### THE CHARITY COMMISSIONERS' PROPOSALS.

Three committees are constituted under the foregoing scheme: managing, hospital, and medical, respectively. Sir E. Schiff is chairman of the managing committee for life, and will appoint his own successor, to hold office for three years. The first co-optative members of the managing committee are the Duchess of Somerset, Miss E. F. Tytler, Mr. E. H. T. Atkinson, Mr. Rowland Berkeley, Mr. W. D. Hoare, Mr. A. B. Lipscomb, Mr. W. McArthur, Mr. Evelyn Morley, Mr. C. F. Pearson, Mr. A. W. Mayo Robson, Mr. Sydney Schiff, and the Rev. F. Whyley; they will hold office for five years, and their successors will be appointed by the managing committee. The hospitals' representative committee is to consist of Mr. W. R. Freeman, Mr. A. H. Campbell, Mr. G. L. Hawker, Mr. W. D. Hoare, Prince Alexander of Teck, Mr. J. G. Wainwright, and Mr. C. F. Pearson. The medical committee is to consist of ten members, one appointed by each of the seven hospitals mentioned, and three by the managing committee. The scheme seems to be a sensible one, and should make for the enhanced success of one of the most useful developments of modern philanthropy.

### FRICTION AT A MENTAL HOSPITAL.

Judging by the last committee meeting at Cardiff Mental Hospital, the food at this institution is still a subject of comment. We previously noted the denial of the medical superintendent, Dr. Goodall, that the food was inferior, as the local branch of the Asylum Workers' Association alleged. We have now to note a petition from the attendants asking for one day's rest in seven for the day staff, and for money in lieu of rations at the rate of 10s. a week while on annual leave, together with further rearrangements and ration allowances. Dr. Goodall pointed out that if the petition were granted it would mean an additional cost of £834, and that to accede to such requests might lead only to their extension. A sub-committee therefore was appointed to inquire into and to report upon the matter. From Dr. Goodall's references to the Trades Council at the same meeting it would seem as if this petition and the preceding complaint are but symptoms of an underlying cause of friction. If so, the sooner it is exposed and dealt with the better.

### THE BOARDING-OUT OF MENTAL PATIENTS.

Dr. H. de Maine Alexander, M.D., medical superintendent of Kingseat Mental Hospital, Aberdeen, has some interesting remarks in his report on the boarding-out of those patients who no longer require institutional treatment. "A man," he reminds us, "who is industrious, quiet, inoffensive, enjoying full liberty at the mental hospital, and just such a man as one would pick out as being eminently suited for boarding-out may, apart altogether from any exacerbation of his mental symptoms, prove an absolute failure in his new abode. I have seen this several times, and a change of guardian led to no better result. Boarding out relieves the ratepayers of building additional accommodation to house those chronic cases who no longer require asylum care, but of course the demand of increased accommodation for acute cases, and for chronic cases requiring supervision, must nevertheless arise coincidently with the rise in the population." This is a very good summary of the possibilities and limitations of the boarding-out system, which Guardians and others without direct administrative experience with difficulty only can be persuaded to recognise.

### HOSPITAL MANAGERS AND THE TERRITORIALS

Among the officers to be Colonels on appointment as Assistant-Directors of Medical Services of Territorials is Lieutenant-Colonel Donald G. Mackintosh, M.V.O., M.B., from the 3rd Scottish General Hospital R.A.M.C., vice Colonel Sir George T. Beatson, K.C.B., M.D., vacated. Dr. Mackintosh is, of course, Medical Superintendent of the Western Infirmary, Glasgow.

### PRESENTATION TO A HOSPITAL CHAIRMAN.

A presentation has been made to Mr. H. H. Hornby, the Chairman of the Liverpool Ear and Eye Infirmary Committee, who has completed fifty years' service as a member of the Committee. Lord Derby, who made the presentation, which was a silver salver suitably inscribed, wished Mr. Hornby many years of health and prosperity. Last year was a record year as regards the work of the in-patient department. There were 1,543 in-patients as compared with 1,213 in 1910. The extra pressure was chiefly due to the greater number of ear cases. Mr. H. H. Hornby, in moving the adoption of the report, pointed out that the work of the Infirmary was satisfactory in one respect, and that was the increasing number of cases dealt with each year. He, however, regretted that the Infirmary was not well supported. In fact, the special appeal issued at the beginning of the year has not met with the response hoped for, and there is still a deficit of £2,740.

### THE SHOPS ACT AND THE PUBLIC HEALTH.

The Shops Act, which came into force on Wednesday, promises to have an important influence on the health of a large section of the working population which is employed in shops. Hitherto legislation affecting the closing of shops has not greatly helped shopkeepers and their assistants, but from now onwards every shop assistant will have a weekly holiday from one o'clock in the afternoon, and, with certain exceptions, the shopkeepers themselves will be obliged to lock up their shops for half a day once a week. Already sports clubs are being formed in many parts of the country by assistants who, until the Act came into force, thought they had played their last game of cricket when they first put on their aprons and went behind the counter. Fres air and exercise for shop assistants, of whom there are little short of a million in Great Britain, mean better health and less medicine; and for that reason, if for no other, those whose desire it is to improve the public health welcome the new legislation.

### HOSPITAL DISPENSERS AND THE INSURANCE ACT.

Some competition has arisen for election to the Council of the Pharmaceutical Society, presumably because of the Insurance Act, which materially affects pharmacists. One of the "planks of the platform" with the new-comers seems to be a promise publicly to advertise all the important posts under the Society, and to give prior consideration to a pharmacist. Mr. Wm. Kirkby, M.Sc., honorary apothecary to the Manchester Infirmary, has addressed a communication to the candidates, which is signed by sixty-four members of the Society, asking for a promise to support this desire. At present important posts, such as Parliamentary Secretary, Local Associations' Officer, and Editor, are now held by pharmacists. We observe that there is no public-institution pharmacist standing for the Council honours, although the Public Pharmacists and Dispensers' Association has the matter under consideration, and hopes next year to have a candidate in the person of a well-known London County Council mental-hospital dispenser. This Association has expressed its displeasure because the Pharmaceutical Standing Committee on Insurance has no representative of the institution dispenser amongst its members. Seeing that the vast experience of this type of pharmacist would have been extremely valuable, this seems a regrettable oversight.

### THE SEX QUESTION IN SCHOOL INSPECTION.

The recent appointment of Dr. Joseph Green, M.D., resident medical officer at the Liverpool Infectious Diseases Hospital, as schools medical officer to the Lancashire Education Committee has created some discussion because the post had previously been held by a woman practitioner. On her retirement the Committee closed the door in advance to all women candidates on the ground that a man "can cover more ground than a woman in the course of a week's work." Presumably the Committee speaks from experience, but we believe that it has been found in many places that school inspection is work which women doctors are well qualified to do, though it is still said by some that a woman makes a better school nurse than a school doctor.

### MRS. KENDAL ON POUND-DAYS.

Mrs. Kendal was in one of her happiest *rôles* last Saturday afternoon, when she opened the eighth annual pound-day in connection with the Belgrave Hospital for Children, Clapham Road. She reminded her hearers that pound-days were not a modern innovation: even in Elizabethan days they had Shylock clamouring for his pound of flesh. The announcement was made that the Beatrice Ward, containing ten beds, is shortly to be reopened. The ward is named after Princess Beatrice, who opened the hospital, and it had to be closed four years ago through lack of funds. The committee has surmounted financial difficulties, and, thanks to Mrs. Bourchier Hawkesley, who has collected over £900, will now be able to utilise to the fullest extent all the hospital premises.

### THE MEDICAL OFFICERS OF HEALTH WEEK.

Where the National Health Week has not been a local affair it has not been a success. We notice, therefore, that medical officers of health have been making the most of it to popularise their teaching in hygiene and sanitation, and to give a common impetus to all local health agencies. Dr. Porter, Medical Officer of Health for Marylebone, as chairman of a committee has been responsible for a full week's programme, which has comprised sermons, lectures, and visits to institutions. If the National Health Week is to become an annual event of any practical value the more enterprising of those who have been responsible for local campaigns on its behalf would do the scheme a service by detailing any results gained by their propaganda. These should be reflected in the dispensaries and other agencies, from the patients who attend which it should soon be learnt what lessons of the many put forward have really been driven home. Even at the end of the week it would be more or less ascertainable on what subjects prejudice or ignorance was most easily broken down. On these points the medical officers of health should have acquired much useful and possibly amusing information, and we invite criticisms of the value of the project.

### EQUIPMENT STARVATION.

A very severe indictment of the equipment at the Devon and Exeter Hospital was uttered by Dr. J. D. Harris last week. "The hospital," he said, "is managed on the most skimpy basis possible. Ridiculous economies have to be made, and the accident-room is absolutely starved. The room and appliances are not anything like sufficient for a hospital of such a size. The staff has not the ordinary appliances which can be found in small hospitals for the detection of difficult cases. Owing to the Governors not having sufficient money the hospital's reputation is endangered. With the present *x*-ray appliances we can detect pins and needles in people's fingers, but that is about all." Alterations, however, for Dr. Harris's department are being considered, and the president, Mr. Tremayne Buller, hopes that some improvement will be made. Modern instruments for *x*-ray work are being constantly demanded up and down the country, and so far it has not generally been difficult to find voluntary givers for them. Devon is one of the very last counties which ought to be slow in providing them.

### THE LINCOLN MEMORIAL.

The recent comments in The Hospital upon the situation with regard to the King Edward Memorial in connection with the Lincoln County Hospital have led Canon Hutton to give an explanation to the local Press. He points out that the recent decision to improve the casualty department without further delay does not reflect upon the King Edward Memorial Committee, because this improvement was not the "essential part" of the memorial scheme. The aim of the memorial was to provide a nurses' home and a women's ward; these two are interdependent, for while further accommodation for patients was asked for by Dr. W. H. Brook and the medical staff, the present nurses' quarters are barely sufficient for the existing number of patients, and so increased accommodation for one must be balanced by enlarged quarters for the other. It was hoped that the improved casualty department might be included in this scheme, though not as an integral part of it. As, however, the memorial fund, which fairly quickly rose to £5,000, has stopped at that sum, the board has decided to take in hand the long-recognised need of a reorganised casualty department. In the meanwhile, presumably, the memorial scheme is at a standstill. It is important, however, to remember that appeals for memorials do not gain in force as the months pass by, and therefore, in spite of the Insurance Act, it is to be hoped that efforts to raise further money at Lincoln will not be wanting.

### FIRE AT AN ISOLATION HOSPITAL.

One-half of the Kingsbury Isolation Hospital was gutted in a serious fire which broke out last week. As there were no patients in the building, which was occupied at the time by the caretaker and his family, who escaped without injury, the fire has attracted little notice. It was, however, sufficiently serious to destroy practically the whole of the left wing, two wards and a receiving room being gutted. The fire, which is said to have been due to the force of the sun, was discovered about nine in the morning, and, though the alarm was at once given, the firemen were not able to abandon their efforts till after one o'clock. We hope that the fact that no life was lost will not hinder a careful investigation into the cause of the outbreak, as such investigations can teach many lessons of which all institutions badly need reminding from time to time.

### SAFETY IN SHIPWRECK.

When the inquiry into the "Titanic" disaster is held in this country, unless the public has exhausted the possibilities of the subject before then, the value of boats to foundered vessels is likely to occupy much of the Commissioners' time. We have, however, signs of the inevitable reaction after the fever of the last fortnight in the letter of a medical man, who himself has survived shipwreck, and who ridicules the idea that a superabundance of boats secures safety. Dr. A. H. Copeman proceeds to describe his experience in 1895 when " in a howling gale " a steamship on which he was travelling ran over a sunken rock on the coast of Australia and sank in about fifteen minutes. Boats, he says, were of such little use then that only one was launched successfully. Dr. Copeman forgets, however, that, accompanied by icebergs or fogs, calm water is just as likely to wreck a vessel as "a howling gale," and that in such emergencies boats are invaluable. For such rough weather as he describes they may well be supplemented with the unsinkable deck-seats which he suggests as substitutes. Have both by all means, but do not forget that ships may founder in water so calm that every boat can be launched without serious danger. All such precautions, however, must be secondary to the avoidance of routes known to be dangerous out of desire to save a few hours on the sea.

### A WELSH COMMITTEE ON HOSPITAL BUSINESS.

The work of building a hospital for the Conway and Penmaenmawr Joint Hospital Board has been delayed during a period of three years, and nobody knows with whom the fault rests. At a recent meeting various attempts were made to put the blame on some one, first on the contractors, then on the clerk, and finally, of course, on the Local Government Board. It was explained that the building was begun before the consent of Mr. Burns' Department had been obtained, or apparently even asked for, and now the architect, Mr. T. B. Farrington, is not willing to state whether the work is finished to his satisfaction or not. So far the expenditure has been £13,225, and the recent discussion achieved nothing beyond an exposure of the Committee's lack of information. The only practical proposal decided was moved by Dr. J. R. Williams, that representatives of each authority, accompanied by the matron, should select the necessary furniture in London. But in the meantime the responsibility for the delays has not been fixed, though it was with this object that the discussion began.

### HOSPITALS AND ROYAL BUSTS.

Mrs. J. T. Ramsden unveiled last week the marble bust of King Edward which she has presented to the West Norfolk and King's Lynn Hospital. It will be remembered that the County Memorial to King Edward has taken the form of an enlargement of the hospital buildings, and it is in a niche over the first landing of the new main staircase that the bust has been placed. The bust, which is the work of Mr. Walter Merritt, is a replica of one which the sculptor has carved for the Guildhall in London. In the many discussions which have taken place upon the King Edward memorials throughout the country there has sometimes been expressed an impatience of any scheme which was not purely practical. The right way is to extend or modify the institution but to have something characteristic, as this bust for instance, to remind posterity in whose honour the improvement was carried out.

### HOSPITALS AND THE COAL STRIKE.

From the experience of the Royal Southern Hospital, Liverpool, it will be seen that the recent coal strike had a serious effect financially upon individual hospitals. At the Royal Southern, notwithstanding that a stock of coal had been accumulated to the full extent of the institution's storing capacity at the commencement of the strike, and that a saving of 54 tons by steam and heating economies during its continuance had been effected, the additional cost amounted to £107. The hospital, however, has not been allowed to suffer, for the whole of this sum has been contributed by an anonymous donor. This gift is worth quoting as reminding other hospitals which have been hard hit by the strike of the value of working out exactly what their financial loss has been. No doubt this has been done in many cases, but where it has not it is hardly too late to invite an act of generosity similar to that enjoyed by the Royal Southern Hospital, Liverpool.

### TEN NEW ENDOWED BEDS AT GLASGOW.

Several of the Glasgow hospitals benefit by the will of the late Mr. William Johnston, a sugar refiner, whose net estate has been valued at over £127,000. The bequests take the form of endowed beds, in memory of the testator's sister, Miss Jemima Johnston. Four are to be endowed at the Western Infirmary, four at the Royal Infirmary, and two at the Victoria Infirmary. It is understood that the bequest is payable at Whit-Sunday 1913. The institutions benefiting, therefore, are not likely to have to wait long before receiving the endowments, which is more satisfactory than when, as of course is frequently the case, the sums allocated depend upon intervening life interests.

### THE POSITION AT THE WEST LONDON HOSPITAL

The Duke of Abercorn, who presided at the annual meeting of the West London Hospital on Tuesday, remarked that the past year was of all their most prosperous, as the result of the progress the institution had made in public esteem. Thus, while the gross income had exceeded that of 1910 by £6,413, the expenditure has remained the same. The year 1911, moreover, began with a debt of £10,247, which has been reduced to about £2,000. It is claimed, therefore, that, if sums temporarily invested during the year are taken into account, the debt is reduced to a lower level than has been the case since 1893. On the other hand, the fund started for a nurses' home for some reason or other has not been well supported. Here, too, as elsewhere, the out-patient department has been outgrown, and much necessary remodelling and enlargement depend upon increased public support. It is to be hoped that next year in this respect also satisfactory progress will be made.

### THE LORD MAYOR AND MENTAL HOSPITALS.

Sir Thomas Crosby, M.D., has been unanimously elected president of the Royal Hospitals of Bridewell and Bethlem, in succession to Sir George Faudel Phillips, who has retired. Sir George had held the position for fifteen years, having been appointed in 1897.

### THE NEW MELBOURNE HOSPITAL.

The foundation-stone of the new buildings in connection with the Melbourne Hospital has been laid with much ceremony. Mr. John Grice, the hospital's president, recalled the circumstance that the new institution was being built upon the old site, and that the estimated cost of buildings and fixtures was £219,000. The amount still required is £50,000 and an additional £12,000 for equipment. In response to a personal appeal for State assistance the State Treasurer, Mr. Watt, virtually promised that that part of the appeal for £60,000 which the public failed to provide the Government would answer for. The great bequest of £120,000 to the building fund from the Edward Wilson trustees was, of course, alluded to. The hospital when finished will claim to be the largest example in the world of the pavilion principle of construction. A front view will disclose three tall blocks connected by corridors, the centre of which is the administrative block, and the two side ones are the surgical and medical wards, with two others of similar design behind them. Each block will be self-contained, with its own theatre. On the left border of the site will be the casualty and out-patient departments, and over the latter will be the nurses' quarters. The hospital was begun in November 1910, and when complete will contain 400 beds.

### THE LATE SIR F. C. WALLIS.

The untimely death of Sir Frederic Wallis removes from among the hospital surgeons of London a striking personality. A breezy manner and a freedom of speech which was sometimes the cause of unintentioned offence did not conceal from those who knew him his kindness of heart and sterling qualities. Sir Frederic, who was knighted only last year, was not one of those men who are so wrapped up in their own particular occupation or profession that they have no time to spare for making themselves useful in other ways or for the social amenities of civilisation. Notwithstanding the faithful discharge of his duties at three hospitals—Charing Cross, St. Mark's, and the Grosvenor—and at several hostels and similar charitable institutions, and the claims of his private patients, he devoted much time to the Union Jack Club, of which he was a staunch supporter. Popular with patients, students, nurses, and in fact with all who knew him, Sir Frederic Wallis will be missed by very many to whom his cheery manner and expressive language have made him a household word. Although a general surgeon of exceptional all-round skill, he was especially adept in the treatment of diseases of the rectum and anus, and had written much upon them. Indeed, a small volume by him upon this subject in a practitioner's series is even now announced for publication. Sir Frederic was educated at Cambridge and at St. Bartholomew's, though he had been for many years on the staff at Charing Cross Hospital. On Tuesday last a memorial service was held at St. Andrew's Church, Wells Street, and was attended by a large number of students and resident medical officers from Charing Cross Hospital, and by all the members of the honorary staff.

### ALTERNATIVES TO THE INSURANCE ACT.

The managers of institutions whose employees now enjoy the benefit of sick pay and medical attendance during illness must shortly consider the question, Is the Act to be adopted or are we to go on much as before? It is a question of greater importance to the servant than to the master; and if it is left to the servants to decide they should weigh the advantages and disadvantages carefully before arriving at a decision. In this connection the proceedings at a meeting of one of the Metropolitan Borough Councils are not without significance. A member of the Council proposed that all the borough employees should be relegated to their rights under the Insurance Act. The suggestion met with violent opposition on the part of the Labour members, all of whom were of opinion that the benefits of the Act were much less favourable to the worker than the existing arrangements of the Council. In the end the consideration of the proposal was adjourned. Managers would do well to make it plain to every member of the staff that the institution cannot afford to pay 3d. a week and continue to provide medical benefit on the present generous scale.

### THIS WEEK'S DRUG MARKET.

The improved tone of the Drug market is well maintained; in fact, if the demand for quinine were a correct barometer of the drug trade, it would be true to describe business as very good. Some large transactions in quinine sulphate have taken place, and a further advance in price is expected; practically everything depends on the result of the sales of cinchona bark to be held in Amsterdam next week, and if, as is anticipated, higher prices are paid for the bark, an advance in the price of quinine is almost inevitable. The price of camphor has been raised by German refiners, but, so far, English refiners have not followed suit. Cod-liver oil again tends rather lower in price, but any substantial fall in value is hardly to be expected in view of the comparatively low figure to which it has already fallen. The Opium market is quiet, with a lower tendency. Balsam copaiba is rather dearer. There has been a slight advance in the price of glycerin, but there is a general feeling that any further substantial advance is not likely to take place at present.

### TO OUR READERS.

Contributions are specially invited from any of our readers to these columns. They should deal with topical subjects and news. They must be authenticated for the information of the Editor only. The minimum payment if published is 5s. There is no hard-and-fast rule as to space, but notes of about twenty lines in length are preferred.

